# Photodynamic therapy (PDT) for oral leukoplakia: a systematic review and meta-analysis of single-arm studies examining efficacy and subgroup analyses

**DOI:** 10.1186/s12903-023-03294-3

**Published:** 2023-08-13

**Authors:** Rui Zhang, Tong Gao, Dan Wang

**Affiliations:** 1 Department of General Stomatology, General Hospital of Ningxia Medical University, Yinchuan, 750004 China; 2 Department of Prosthodontics, Affiliated Hospital of Yanan University, Shaanxi, 716000 China; 3 Department of Stomatology, Qingtongxia Hospital of Traditional Chinese Medicine, Ningxia, 751600 China

**Keywords:** Photodynamic therapy, Oral leukoplakia, meta-analysis, Single arm, Subgroup analysis

## Abstract

**Objective:**

This study aims to evaluate the efficacy of photodynamic therapy (PDT) in the treatment of oral leukoplakia and explore the subgroup factors that may influence its effectiveness.

**Methods:**

A systematic search was conducted in PubMed, Embase, the Cochrane Library, and Web of Science databases to identify relevant studies. Meta-analysis was performed using Stata15.0 software. Cochran’s Q test and I^2^ statistics were used to evaluate heterogeneity, egger’s test was used to evaluate publication bias.

**Results:**

The analysis of 17 studies included in this study suggests that PDT may be effective in achieving complete response (CR) [ES = 0.50, 95%CI: (0.33,0.66)], partial response (PR) [ES = 0.42, 95%CI: (0.27,0.56)], no response (NR) [ES = 0.19, 95%CI: (0.11,0.27)]in patients with oral leukoplakia. The recurrence rate was also evaluated [ES = 0.13, 95%CI: (0.08,0.18)]. Subgroup analysis showed that various factors such as light source, wavelength, medium, duration of application, clinical and pathological diagnosis classification influenced efficacy of PDT. The lesion areas of the leukoplakia after treatment were reduced by 1.97cm^2^ compared with those before treatment.

**Conclusion:**

Our findings show that PDT is a viable treatment for oral leukoplakia. However, the effectiveness of the therapy may depend on several factors, as suggested by our subgroup analyses. (Registration no. CRD42023399848 in Prospero, 26/02/2023)

**Supplementary Information:**

The online version contains supplementary material available at 10.1186/s12903-023-03294-3.

## Introduction

Leukoplakia is a white lesion in the oral mucosa, excluding other white lesions that can be diagnosed clinically, histopathologically, and by auxiliary means, and is usually non-erasable [1]. The etiology of leukoplakia is not fully understood, but chronic local irritation, smoking, and areca nut chewing are considered possible causes. Oral leukoplakia is a common and potentially malignant oral disease, with a high risk of progressing to squamous cell carcinoma. The global incidence of oral leukoplakia is 4.11% [2]. Clinicopathological and systematic review studies indicate that oral leukoplakia’s malignant transformation rate is 7.5% and 9.7%, respectively. In clinical studies, although some cases of leukoplakia had clinically benign features, some parts of the tissues were found to have transformed into malignant lesions by further histopathological examination [3, 4]. Since leukoplakia is a precancerous lesion that can cause systemic health effects if left untreated, certain studies have documented its associations with an increased risk of upper gastrointestinal cancers [5, 6]. Therefore, prompt treatment of oral leukoplakia is critical. Traditional methods for treating leukoplakia include systemic drug application and local surgical excision. However, these therapies have significant drawbacks, including drug side effects and tissue defects after surgery. Therefore, cryotherapy, laser, and photodynamic therapy (PDT) have become increasingly common in clinical practices [7, 8].

PDT is a minimally invasive treatment that uses exogenous light and photosensitizers to sensitize tumor tissue to specific light wavelengths. Activation of photosensitizers in tissues by these wavelengths creates reactive oxygen species (ROS) by transferring energy from the light to molecular oxygen [9, 10]. The destruction of tumors mediated by PDT occurs through three main mechanisms. Firstly, ROS directly kill tumor cells. Secondly, PDT can disrupt the vascular system associated with the tumor, leading to thrombosis and subsequent tumor infarction. Finally, PDT can lead to an immune response against tumor cells [9]. PDT has several advantages over traditional treatments: it is less invasive, causes fewer side effects than systemic medication, and is more precise in targeting the lesion while preserving normal tissues. Nowadays, PDT therapy has been widely used to treat oral diseases, including leukoplakia [11–14]. However, existing reports differ in the types of medium, duration of application, light source, and wavelength used, among other factors. There is no standard reference for clinical practice. Therefore, this study aims to conduct a meta-analysis on the efficacy of PDT in treating oral leukoplakia, comparing various factors that may influence its effectiveness. The results of this study are expected to guide clinical practice.

## Materials and methods

This systematic review was reported according to the Preferred Reporting Items for Systematic Reviews and Meta-Analyses (PRISMA) 2020 statement. The detailed PRISMA checklist shown in Additional File 1 Table S1.

### Search strategy

We comprehensively searched the PubMed, Embase, the Cochrane Library, and Web of Science databases from inception to January 29, 2023. The search keywords were “Leukoplakia, Oral” and “Photochemotherapy”. The search strategy includes subject terms and free words. The specific search strategy in PubMed was as follows: (((“Photochemotherapy“[MeSH Terms] OR (“Photochemotherapy“[Title/Abstract] OR “Photochemotherapies“[Title/Abstract])) AND ((“leukoplakia, oral“[MeSH Terms] OR (“leukoplakia oral“[Title/Abstract] OR “leukoplakias oral“[Title/Abstract] OR “oral leukoplakia“[Title/Abstract]))). We did not place any restrictions on language, study type, or format to ensure the integrity of the search. The details of literature retrieval are recorded in Table S2 (see Additional File 2).

### Inclusion and exclusion criteria

The inclusion criteria were applied: (1) Prospective and retrospective single arm clinical studies; (2) Studies that included patients diagnosed with leukoplakia, regardless of the classification of leukoplakia, based on pathological diagnosis and clinical diagnosis; (3) Studies that involved the treatment of PDT, either as a standalone therapy or in combination with other methods; (4) Outcome measures included complete response (CR), partial response (PR), no response (NR), and recurrence (Recurrence). (5) Studies with overlapping populations of the same author only use the most recent studies.

The exclusion criteria were applied: (1) Meeting minutes, review articles, study design methods, case reports, correspondence, and basic and animal experiments; (2) Reports on multiple population or disease cohort; (3) Study that did not involve the use of PDT; (4) Studies without valid data.

### Data extraction

The two researchers independently screened the literature by reading the titles and abstracts of the studies and excluded irrelevant articles. They then reviewed e full-text articles to determine if they met the inclusion criteria and extracted relevant data. Any disagreements were resolved through discussion, with the involvement of a third researcher when necessary, to ensure consistency in the selection of the study and data extraction. Data collected from the included studies included the first author, year of publication, country, participants, number of lesions, gender and age of participants, size of lesion, pathological diagnosis, clinical classification, intervention (light source, wavelength, medium, duration of application), outcome index (CR, PR, NR, Recurrence), and side effects.

### Quality assessment

The quality assessment of adopted non-randomized controlled studies (single arm studies) was conducted using Methodological Index for Non-randomized Studies (MINORS) [15]. The MINORS tool evaluates the following items: (1) A clearly stated aim; (2) Inclusion of consecutive patients; (3) Prospective collection of data; (4) Endpoints appropriate to the aim of the study; (5) Unbiased assessment of the study endpoint; (6) Appropriate follow-up period the study aim; (7) Loss to follow up less than 5%; (8) Prospective calculation of the study size. Each item is scored 0 (not reported), 1 (reported but inadequate) or 2 (reported and adequate).

### Statistical analysis

In this study, Stata15.0 software was used to perform statistical analysis of the effect size (ES) and 95% confidence interval (CI) for outcomes of complete response (CR), partial response (PR), no response (NR), and Recurrence in patients with oral leukoplakia after PDT treatment. Subgroup analysis was conducted based on different media types, light sources, and other factors. Measurement datas were calculated using weighted mean difference (WMD) and 95% CI. Heterogeneity was evaluated using Cochran’s Q test and I^2^ statistics with I^2^ values of 0%, 25%, 50%, and 75% indicating no, low, medium, and high heterogeneity, respectively. A random effects model was used when I^2^ ≥ 50%, and sensitivity analysis was performed to explore possible sources of heterogeneity. A fixed effects model was used when I^2^ < 50%. Publication bias was assessed using Egger’s test.

## Results

### Study selection

**Fig. 1:**
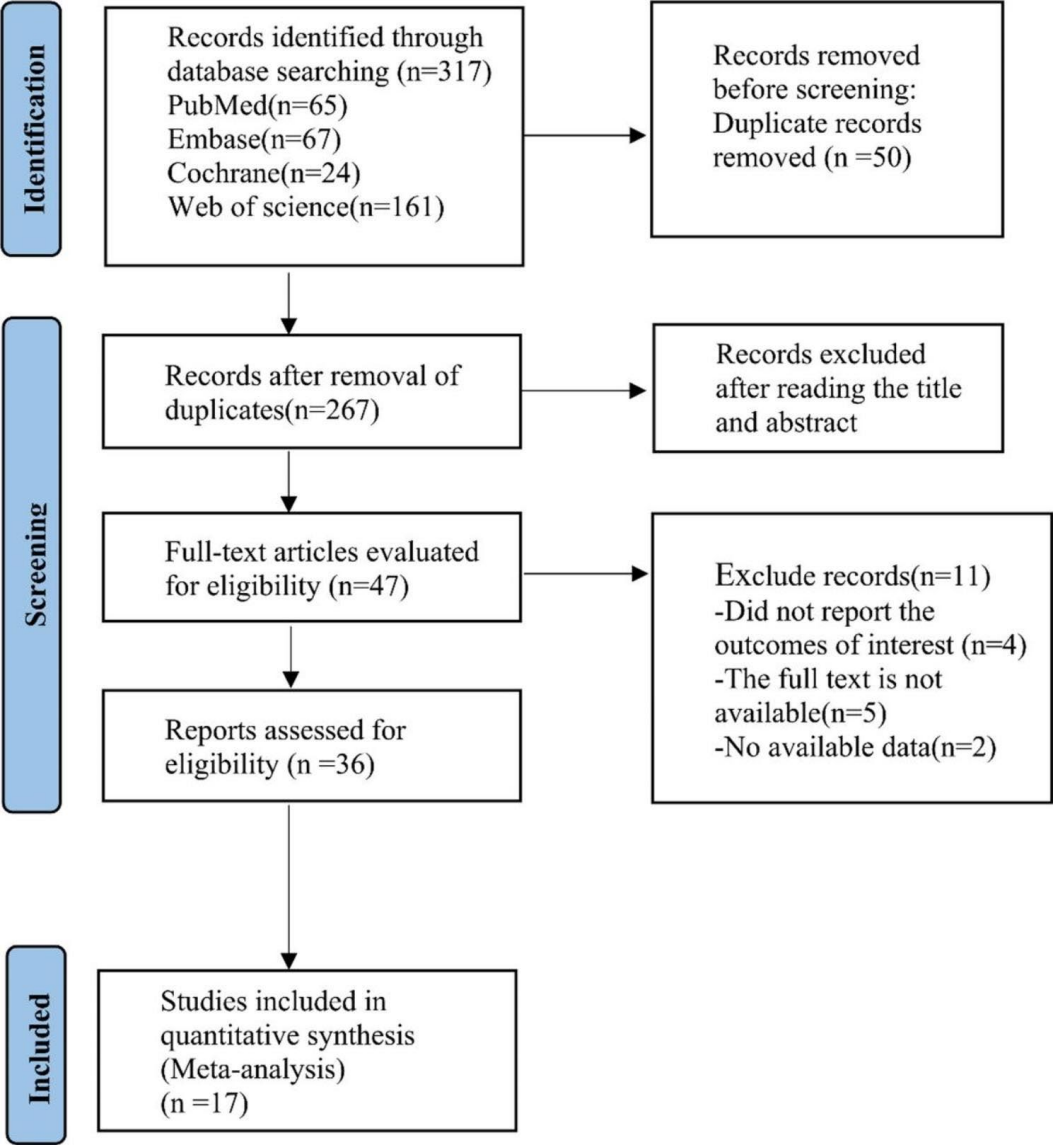
Flow diagram of the literature retrieval, seventeen studies were eventually included

Initially, we screened 317 relevant studies from various databases, including 65 from PubMed, 67 from Embase, 24 from Cochrane, and 161 from Web of science. After removing duplicates and excluding irrelevant studies, we included 17 single arm studies [16–32] in the final analysis. The flow chart detailing the literature retrieval process is presented in Fig. 1.

### Characteristics of the included study

A total of 17 single-arm studies were included in this analysis, there were 16 studies in English and 1 study [27] in Russian, involving 662 participants and 702 diagnosed leukoplakia lesions treated with PDT. The basic characteristics of the included studies are shown in Table S3 (see Additional File 3).

### Quality assessment

The 17 single-arm studies were evaluated for quality using the MINORS criteria. Seven studies scored more than 9 points [19–21, 24, 26, 28, 32]. Eight studies scored equal to 9 [16, 17, 22, 23, 27, 29, 30, 31], and two studies scored less than 9 [18, 25].

### Results of meta-analysis

#### Analysis result of CR outcome

**Fig. 2:**
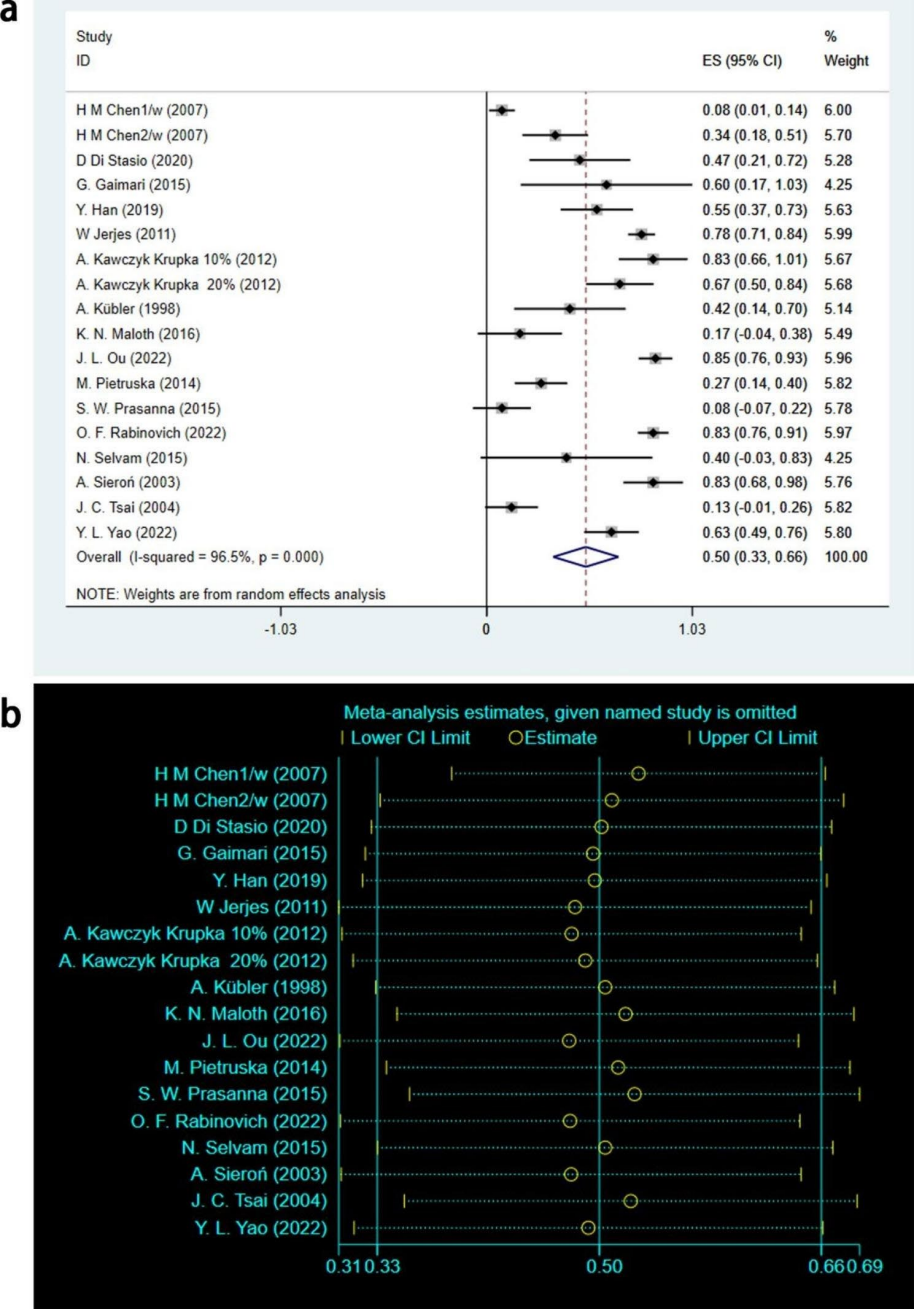
CR outcomes of meta-analysis. **(a)** Forest plot. ES means effect size; CI means confidence interval. **(b)** Heterogeneity analysis diagram. In these studies, no clearly hererogeneous origin could be found

Of the 17 included studies, 16 reported CR outcomes involving 401 leukoplakia lesions [16–28, 30–32]. The statistical analysis of CR outcome showed that [ES = 0.50, 95%CI: (0.33,0.66), I^2^ = 96.5%, *P* < 0.001]. A random effects model was used for the analysis, as shown in Fig. 2a. Further sensitivity analysis was performed to evaluate heterogeneity and the stability of the CR outcome, as shown in Fig. 2b. The analysis showed that the sensitivity was low and the results were stable. The overall analysis revealed that 50% of leukoplakia lesions achieved complete remission after PDT treatment, which was statistically significant.

#### Analysis result of PR outcome

**Fig. 3:**
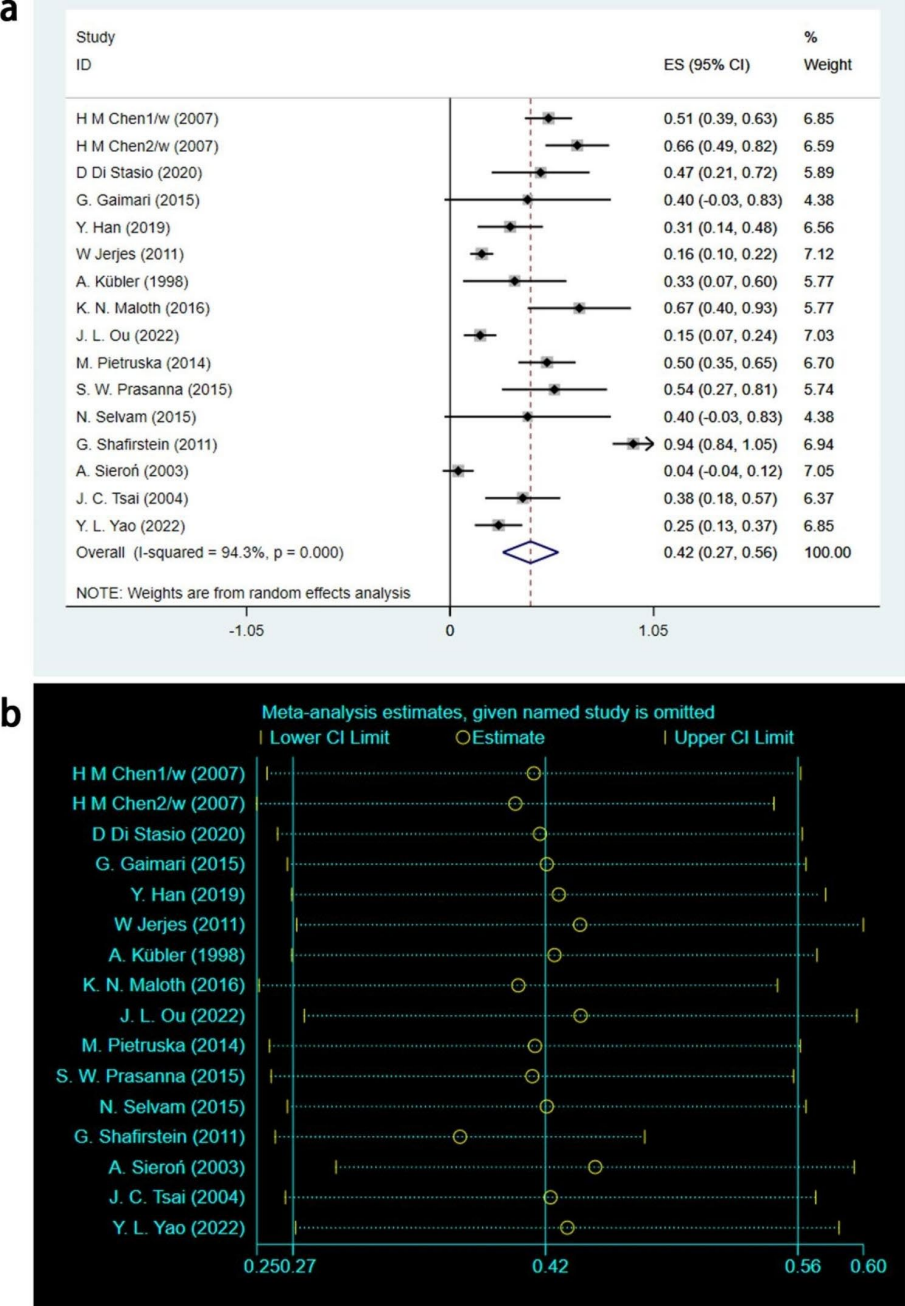
PR outcomes of meta-analysis. **(a)** Forest plot. ES means effect size; CI means confidence interval. **(b)** Heterogeneity analysis diagram. In these studies, no clearly hererogeneous origin could be found

A total of 15 studies reported PR outcomes involving 189 leukoplakia lesions [16–20, 22–26, 28–32]. After statistical analysis using the random effects model, it was concluded that [ES = 0.42, 95%CI: (0.27, 0.56), I^2^ = 94.3%, P < 0.001], as depicted in Fig. 3a. Sensitivity analysis was conducted to evaluate the heterogeneity of PR outcome, as illustrated in Fig. 3b. The analysis indicated low sensitivity and good stability. The findings of this study revealed that 42% of leukoplakia lesions showed partial improvement after PDT treatment, and the results were statistically significant.

#### Analysis result of NR outcome

**Fig. 4:**
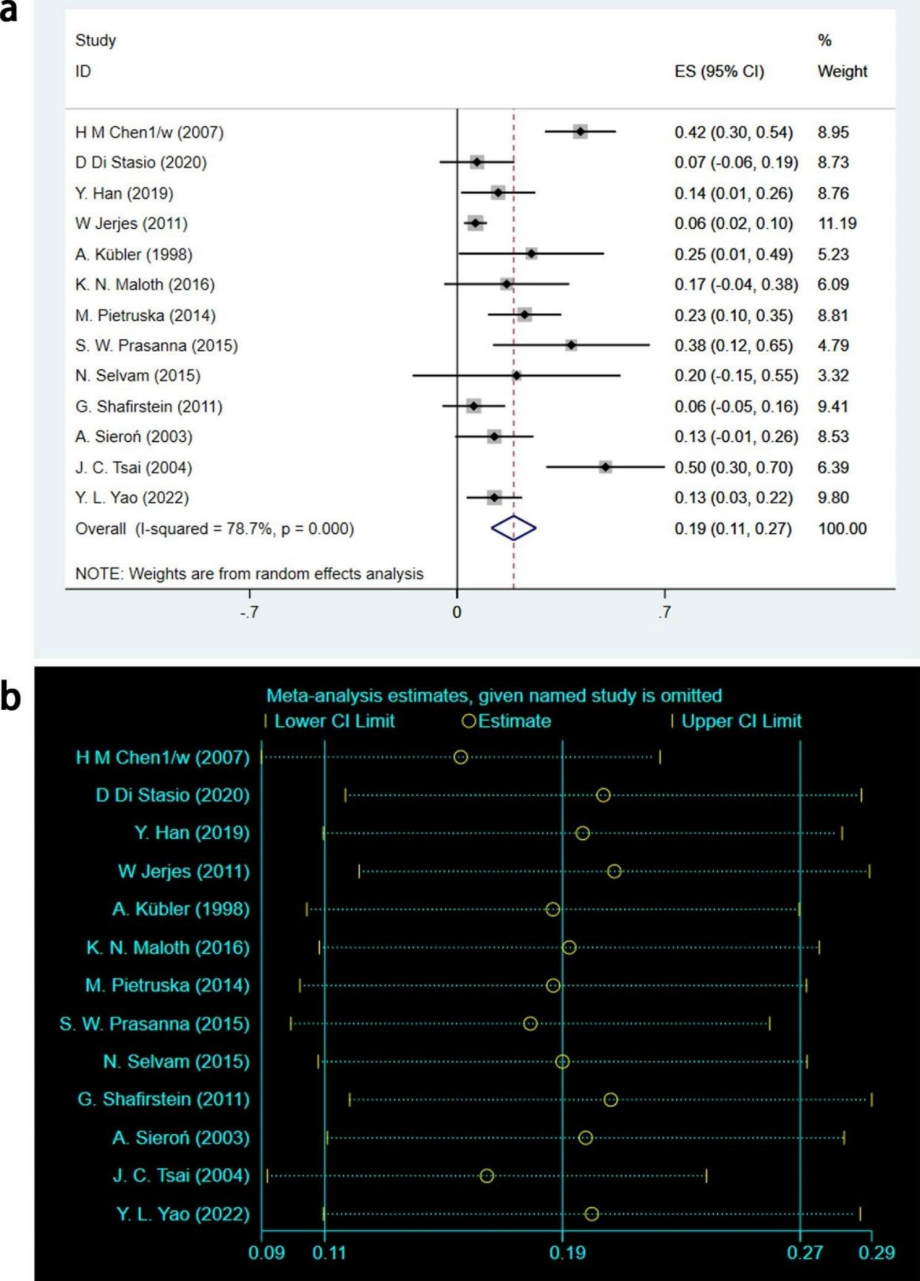
NR outcomes of meta-analysis. **(a)** Forest plot. ES means effect size; CI means confidence interval. **(b)** Heterogeneity analysis diagram. In these studies, no clearly hererogeneous origin could be found

A total of 13 studies involving 84 leukoplakia lesions [16, 17, 19, 20, 22, 23, 25, 26, 28–32] with NR outcomes were analyzed. After statistical analysis using the random effects model, it was concluded that [ES = 0.19, 95%CI: (0.11, 0.27), I^2^ = 78.7%, P < 0.001], as shown in Fig. 4a. The sensitivity analysis was conducted for the NR outcome heterogeneity, and the results indicated a small sensitivity with good stability, as shown in Fig. 4b. The test showed that 19% of leukoplakia did not resolve after PDT treatment, and the results were statistically significant.

#### Analysis result of Recurrence outcome

**Fig. 5:**
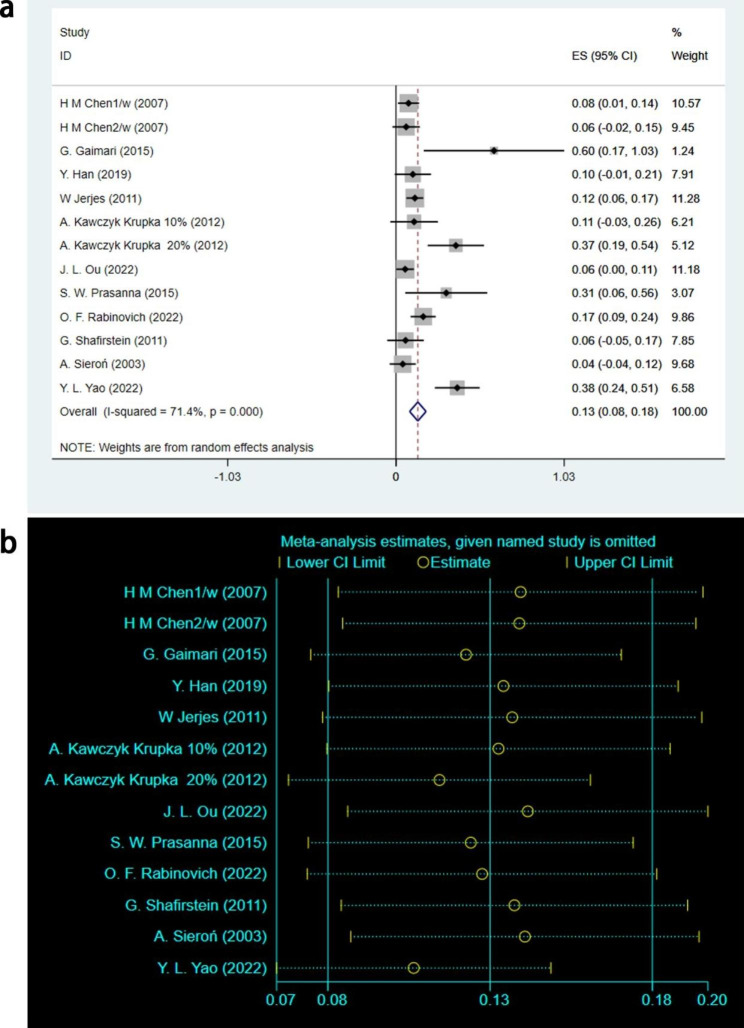
Recurrence outcomes of meta-analysis. **(a)** Forest plot. ES means effect size; CI means confidence interval. **(b)** Heterogeneity analysis diagram. In these studies, no clearly hererogeneous origin could be found

A total of 11 studies involving 84 leukoplakia lesions [16, 18–21, 24, 26, 27, 29, 30, 32] with Recurrence outcomes were analyzed. After statistical analysis using the random effects model, it was concluded that [ES = 0.13, 95%CI: (0.08, 0.18), I^2^ = 71.4%, *P* < 0.001], as shown in Fig. 5a. The sensitivity analysis was conducted for the Recurrence outcome heterogeneity, and the results indicated a small sensitivity with good stability, as shown in Fig. 5b. The test results showed that 13% of leukoplakia lesions recurred during follow-up after PDT treatment, and the results were statistically significant.

### Subgroup analysis

#### CR outcomes

##### According to the influencing factors

Subgroup analysis of complete remission (CR) outcome in PDT treatment for leukoplakia was presented in Table 1 (Forest plots see Additional File 4 Figure S1). The analysis considers the following factors: light source, medium, wavelength, and duration of application.

**Table 1:** Subgroup analysis outcomes of CR in PDT treatment for leukoplakia

Subgroup		NO. of study	Heterogeneity test		ES (95% CI)
			I^2^%	*P*	
Light source	LED	7	98%	< 0.001	0.44 (0.16,0.71)
	Laser	7	87.7%	< 0.001	0.63 (0.47,0.79)
	Other	2	48.8%	0.162	0.17 (-0.12,0.46)
Medium	10% ALA	3	45.4%	0.160	0.78 (0.62,0.94)
	20% ALA	8	96.7%	< 0.001	0.47 (0.22,0.71)
	Other	6	96.6%	< 0.001	0.44 (0.18,0.70)
Wavelength	≤ 640 nm	13	95.8%	< 0.001	0.46 (0.28,0.64)
	＞640 nm	3	96.7%	< 0.001	0.66 (0.36,0.95)
Duration of application	≤ 2 h	11	96%	< 0.001	0.41 (0.22,0.61)
	> 2 h	5	62.7%	0.03	0.76 (0.67,0.85)
Pathologic diagnosis	No dysplasia	4	89.6%	< 0.001	0.51 (0.28,0.73)
	Mild dysplasia	6	0%	0.986	0.12 (0.08,0.16)
	Moderate dysplasia	3	0%	0.984	0.18 (0.13,0.24)
	Severe dysplasia	2	88.6%	0.003	0.22 (-0.03,0.48)
Clinical classification	Homogeneous	2	0%	0.364	0.44 (0.29,0.59)
	Non-homogeneous	2	0%	0.877	0.07 (-0.01,0.15)

In terms of the light source used in the studies, seven studies used laser-emitting diodes (LED) [16, 17, 23, 24, 27, 31, 32], seven studies used laser [18–22, 25, 30], two studies used other light sources [26, 28]. The results of LED and laser test were as follows: [I^2^ = 98%, *P* < 0.001, ES = 0.44, 95%CI: (0.16, 0.71)] and [I^2^ = 87.7%, *P* < 0.001, ES = 0.63, 95%CI: (0.47, 0.79)], indicating that both LED and laser as the light sources for PDT treatment of leukoplakia achieved complete remission of the lesions. However, the remission rate of laser was higher than that of LED.

Regarding the medium used, three studies used 10% aminolevulinic acid (ALA) [21, 28, 30], eight studies used 20% ALA [16, 18, 19, 21, 22, 24, 31, 32], six studies used other mediums [17, 20, 23, 25–27]. The results of the 10% ALA and 20% ALA test were as follows: [I^2^ = 45.4%, *P* = 0.16, ES = 0.78, 95%CI: (0.62, 0.94)] and [I^2^ = 96.7%, *P* < 0.001, ES = 0.47, 95%CI: (0.22, 0.71)], indicating that using 10% ALA as a medium for PDT treatment was more effective for complete remission of lesions.

In terms of wavelength, 13 studies used ≤ 640 nm [16–23, 26, 28, 30–32], and three studies used > 640 nm [24, 25, 27]. The results of ≤ 640 nm and > 640 nm test were as follows: [I^2^ = 95.8%, *P* < 0.001, ES = 0.46, 95%CI: (0.28, 0.64)] and [I^2^ = 96.7%, *P* < 0.001, ES = 0.66, 95%CI: (0.36, 0.95)]. The results showed that PDT treatment was more effective for complete remission when the wavelength was > 640 nm.

Among the duration of the application subgroup, 11 studies used ≤ 2 h [16–19, 21–23, 25–27, 31], and five studies used > 2 h [20, 24, 28, 30, 32]. The results of the ≤ 2 h and > 2 h test were as follows: [I^2^ = 96%, *P* < 0.001, ES = 0.41, 95%CI: (0.22, 0.61)] and [I^2^ = 62.7%, *P* = 0.03, ES = 0.76, 95%CI: (0.67, 0.85)], respectively. The results indicated that PDT treatment was more effective in achieving complete remission of lesions when the duration of application was longer than two hours.

##### According to the classification of pathological diagnosis and clinical

**Fig. 6:**
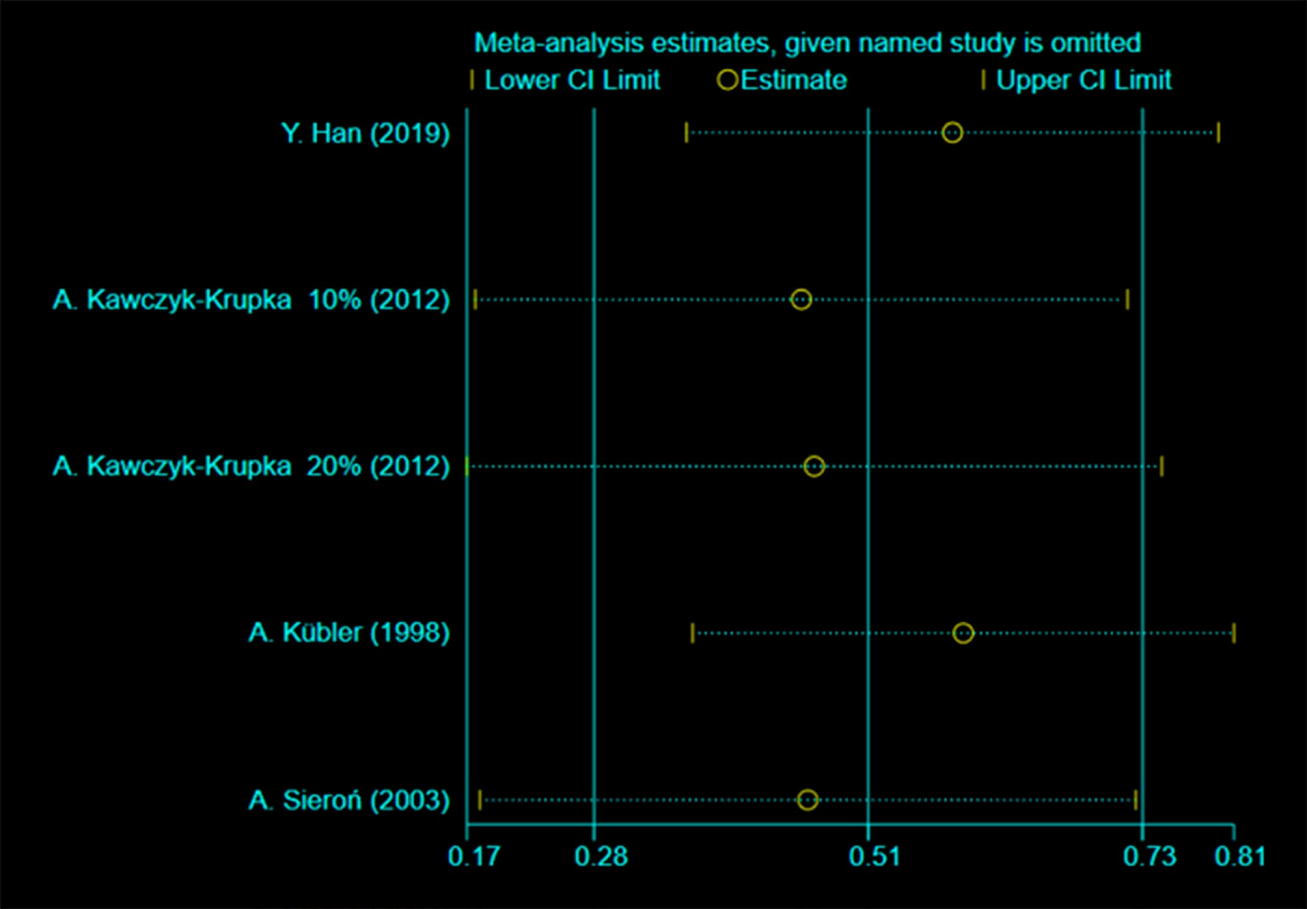
Heterogeneity analysis diagram of no dysplasia. In these studies, no clearly hererogeneous origin could be found

Pathological diagnosis was classified into four categories: no dysplasia (four studies) [19, 21, 22, 30], mild dysplasia (six studies) [19–22, 28, 30], moderate dysplasia (three studies) [19, 20, 28], and severe dysplasia (two studies) [20, 22]. The results showed that PDT treatment had a significant complete remission effect on leukoplakia diagnosed as no dysplasia, mild dysplasia, and moderate dysplasia, with complete remission rates of 51%, 12%, and 18%, respectively. The details are no dysplasia [I^2^ = 89.6%, *P* < 0.001, ES = 0.51, 95%CI: (0.28, 0.73)], mild dysplasia [I^2^ = 0%, *P* = 0.986, ES = 0.12, 95%CI: (0.08, 0.16)], moderate dysplasia [I^2^ = 0%, *P* = 0.984, ES = 0.18, 95%CI: (0.13, 0.24)], severe dysplasia [I^2^ = 88.6%, *P* = 0.003, ES = 0.22, 95%CI: (-0.03,0.48)], respectively presented in Table 1 (Forest plots see Additional File 4 Figure S2). Heterogeneity was high in the no dysplasia and severe dysplasia categories, with I^2^ > 50%, and the random effects model was used. A fixed effects model was used for the other diagnoses. No dysplasia heterogeneity was further analyzed for sensitivity, with low sensitivity and good stability, as shown in Fig. 6.

Regarding clinical classification, two studies categorized the leukoplakia as homogeneous [19, 22], and two studies classified the leukoplakia as non-homogeneous [19, 22]. The results of the tests on homogeneous and non-homogeneous leukoplakia were as follows: [I^2^ = 0%, *P* = 0.364, ES = 0.44, 95%CI: (0.29, 0.59)] and [I^2^ = 0%, *P* = 0.877, ES = 0.07, 95%CI: (-0.01, 0.15)]. These results indicated that PDT treatment was more effective for complete remission when the leukoplakia was clinically classified as homogeneous. (Forest plots see Additional File 4 Figure S3)

#### PR outcomes

##### According to the influencing factors

Subgroup analysis was conducted to evaluate the effect of PDT treatment for leukoplakia PR based on different influencing factors, including the light source, medium, wavelength, and duration of application, as shown in Table 2 (Forest plots see Additional File 4 Figure S4).

**Table 2:** Subgroup analysis outcomes of PR in PDT treatment for leukoplakia

Subgroup		NO. of study	Heterogeneity test		ES (95% CI)
			I^2^%	*P*	
Light source	LED	6	87.7%	< 0.001	0.43 (0.26,0.59)
	Laser	7	97.1%	< 0.001	0.38 (0.12,0.65)
	Other	2	0%	0.593	0.50 (0.27,0.73)
Medium	10% ALA	2	61.3%	0.108	0.16 (-0.17,0.48)
	20% ALA	8	94.7%	< 0.001	0.44 (0.23,0.65)
	Other	5	88.8%	< 0.001	0.45 (0.23,0.68)
Wavelength	≤ 640 nm	13	94.7%	< 0.001	0.43 (0.26,0.60)
	＞640 nm	2	93.7%	< 0.001	0.32 (-0.02,0.66)
Duration of application	≤ 2 h	10	85.3%	< 0.001	0.53 (0.38,0.67)
	> 2 h	5	63.6%	0.027	0.15 (0.08,0.23)
Pathologic diagnosis	No dysplasia	4	0%	0.432	0.08 (0.04,0.13)
	Mild dysplasia	2	21.2%	0.260	0.15 (0.04,0.26)
	Moderate dysplasia	2	0%	0.573	0.04 (-0.02,0.10)
Clinical classification	Homogeneous	2	12.4%	0.285	0.13 (0.03,0.23)
	Non-homogeneous	2	21.2%	0.260	0.15 (0.04,0.26)

Regarding the light source, six studies used LED [16, 17, 23, 24, 31, 32], seven studies used laser [18–20, 22, 25, 29, 30], and two studies used other light sources [26, 28]. The results of LED and laser test were as follows: [I^2^ = 87.7%, *P* < 0.001, ES = 0.43, 95%CI: (0.26, 0.59)] and [I^2^ = 97.1%, *P* < 0.001, ES = 0.38, 95%CI: (0.12, 0.65)], respectively. LED and laser were found to be in achieving partial remission of the leukoplakia lesions, with LED showing a slightly higher partial remission rate than laser.

Regarding the medium, two studies used 10% ALA [28, 30], eight studies used 20%ALA [16, 18, 19, 22, 24, 29, 31, 32], and five studies used other mediums [17, 20, 23, 25, 26]. The results of the 10% ALA and 20% ALA test showed [I^2^ = 61.3%, *P* = 0.108, ES = 0.16, 95%CI: (-0.17, 0.48)] and [I^2^ = 94.7%, *P* < 0.001, ES = 0.44, 95%CI: (0.23, 0.65)], respectively. Therefore, using 20% ALA as a medium for PDT treatment was more effective for partial remission of leukoplakia lesions.

Regarding the wavelength, 13 studies used ≤ 640 nm [16–20, 22, 23, 26, 28–32], and two studies used > 640 nm [24, 25]. The results of the ≤ 640 nm and > 640 nm test showed [I^2^ = 94.7%, *P* < 0.001, ES = 0.43, 95%CI: (0.26, 0.60)] and [I^2^ = 93.7%, *P* < 0.001, ES = 0.32, 95%CI: (-0.02, 0.66)]. PDT treatment was more effective in achieving partial remission when the wavelength was ≤ 640 nm.

Regarding the duration of application, ten studies applied PDT for ≤ 2 h [16–19, 22, 23, 25, 26, 29, 31], and five studies applied > 2 h [20, 24, 28, 30, 32]. The results of ≤ 2 h and > 2 h showed [I^2^ = 85.3%, *P* < 0.001, ES = 0.53, 95%CI: (0.38, 0.67)] and [I^2^ = 63.6%, *P* = 0.027, ES = 0.15, 95%CI: (0.08, 0.23)], respectively. PDT treatment was more effective in achieving partial remission of leukoplakia lesions when the duration of application was less than two hours.

##### According to the classification of pathological diagnosis and clinical

Regarding the classification of the pathological diagnosis, there were four studies on no dysplasia [19, 21, 22, 30], two studies of mild dysplasia [19, 22], and two studies of moderate dysplasia [19, 22]. The test results showed no dysplasia [I^2^ = 0%, *P* = 0.432, ES = 0.08, 95%CI: (0.04, 0.13)], mild dysplasia [I^2^ = 21.2%, *P* = 0.260, ES = 0.15, 95%CI: (0.04, 0.26)], moderate dysplasia [I^2^ = 0%, *P* = 0.573, ES = 0.04, 95%CI: (-0.02, 0.10)], respectively in Table 2 (Forest plots see Additional File 4 Figure S5). I^2^ < 50% was used in the fixed effect model analysis. It is concluded that partial remission rates for no dysplasia and mild dysplasia were 8% and 15%, respectively.

Regarding clinical classification, two studies classified the leukoplakia as homogeneous [19, 22], and two studies classified the leukoplakia as non-homogeneous [19, 22]. The results of the tests on homogeneous and non-homogeneous leukoplakia were as follows: [I^2^ = 12.4%, *P* = 0.285, ES = 0.13, 95%CI: (0.03, 0.23)] and [I^2^ = 21.2%, *P* = 0.260, ES = 0.15, 95%CI: (0.04, 0.26)]. The results showed that the partial remission effect of PDT on homogeneous and non-homogeneous leukoplakia was similar. (Forest plots see Additional File 4 Figure S6)

#### Outcome of changes in the size of leukoplakia after PDT treatment

In terms of the size of leukoplakia lesions, 69 lesions from 3 studies were examined [23, 25, 26]. The control group consisted of the Mean ± standard deviation (SD) before treatment, while the experimental group consisted of the Mean ± SD after PDT treatment. Changes in the lesion size were analyzed through continuous variables. Results showed a statistically significant difference [I^2^ = 82.8%, *P* = 0.003, WMD=-1.97, 95%CI: (-3.51, -0.43)], indicating that after treatment, the lesion areas of leukoplakia were reduced by 1.97cm^2^ compared to before treatment. (Forest plots see Additional File 4 Figure S7a) Heterogeneity was further analyzed for sensitivity, with low sensitivity and good stability, as shown in Additional File 4 Figure S7b.

### Publication bias

**Fig. 7:**
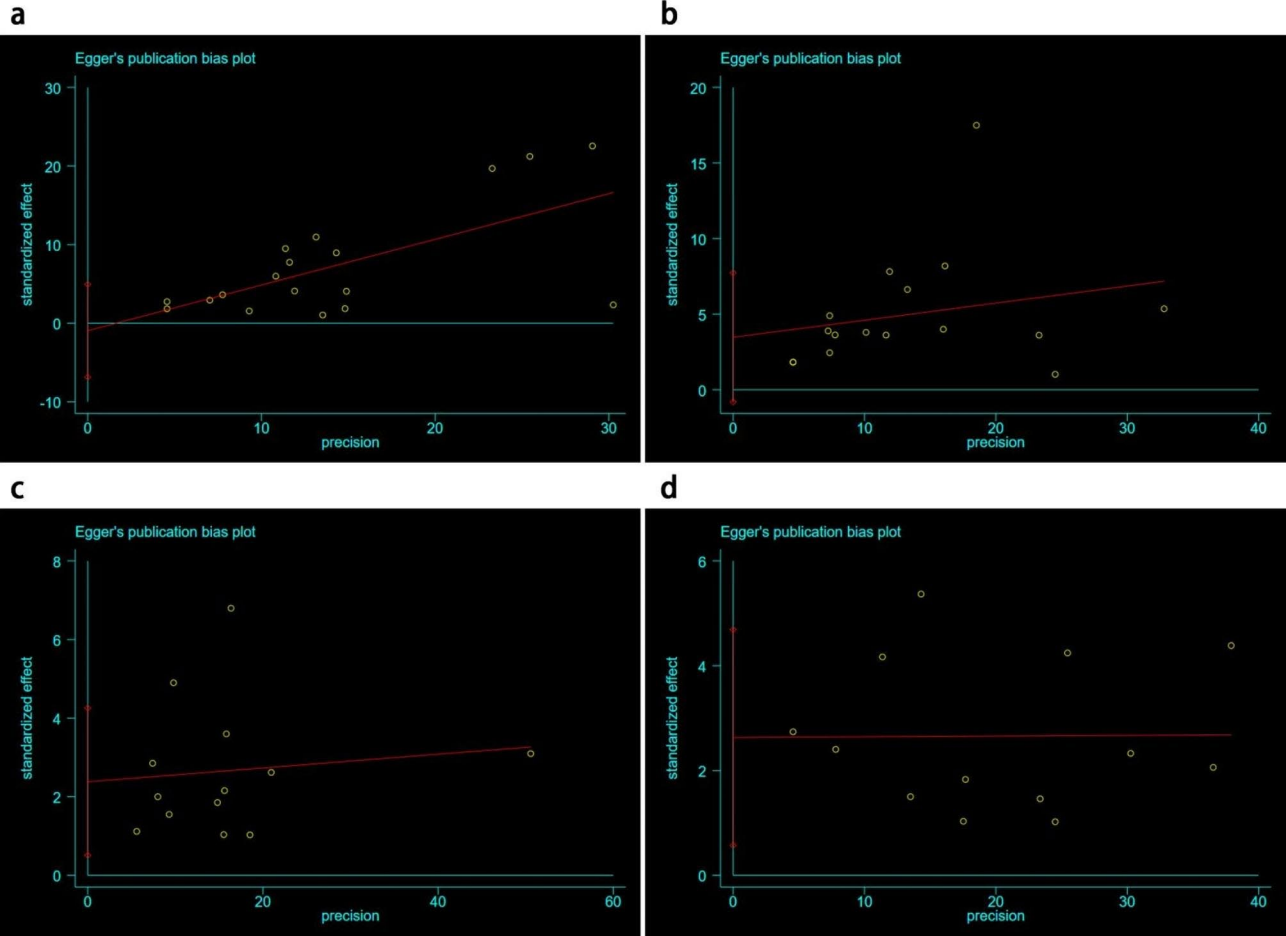
Egger’s publication bias plots. **(a)** CR test. **(b)** PR test. **(c)** NR test. **(d)** Recurrence test

In this study, Egger test was utilized to examine the publication bias of the articles. The result showed no significant publication bias observed in CR and PR since *P* = 0.737 and *P* = 0.103 after conducting the CR and PR tests. However, NR and Recurrence have publication bias since both *P* = 0.017 after conducting tests. (See Fig. 7)

## Discussion

In this study, a meta-analysis was conducted to evaluate the effectiveness of PDT therapy in the treatment of oral leukoplakia. Our results revealed PDT therapy led to complete response in 50% of cases, partial response in 42% of cases, no response in 19% of cases, and recurrence in 13% of cases. The lesion areas of the leukoplakia after treatment were reduced by 1.97cm^2^ compared with those before treatment. These findings were consistent with the conclusions drawn in the systematic review published by Li et al., where the complete and partial response rates were reported to be 32.9% and 43.2%, respectively, and the recurrence rate was below 20% [33]. Notably, the complete response rate reported was lower than that in our analysis, which could be attributed to differences in the number of studies, including the number of participants or lesions and the statistical algorithm employed.

PDT therapy consists of three main elements: a photosensitizer, a light source with specific wavelength, and molecular oxygen [34]. The most commonly used photosensitizer is 5-aminolevulinic acid (5-ALA) or its ester, methy aminolevulinate (MAL) [35]. ALA is not a photosensitizer, but a biological precursor of protoporphyrin IX (PpIX). Under a specific wavelength, PpIX is activated and transmits energy to molecular oxygen, forming reactive oxygen species (ROS), which can cause cell damage or form thrombus in the blood vessels of the injured site, ultimately achieving a therapeutic effect [36, 37]. In the included studies, 13 used 5 - ALA [16, 18–24, 28–32]. The complete remission rate of 10% ALA reached 78%, and the total complete and partial response rate of 20% ALA reached 91%, indicating that 5-ALA is a very effective adjuvant medium in PDT treatment. Stasio et al. used toluidine blue as photosensitizer in their clinical application. They believed that compared with 5-ALA, toluidine blue has the advantages of long duration of light effect, simple operation, lower cost, and no reported side effects [17]. However, due to the small sample size, further clinical studies are necessary to verify whether toluidine blue is superior to 5-ALA. This study found that when the duration of application time was greater than two hours, the CR reached 76%. Conversely, the PR was observed to be relatively high when the duration of application was less than or equal to two hours, which may be attributed to the drug’s penetration depth. As the duration of the application increases, the drug penetrates deeper into the tissues, resulting in a more sensitive tissue response to irradiation.

In PDT therapy, common light sources include lasers, LED, and incandescent light [38]. Among the studies included in this paper, eight used laser [18–22, 25, 29, 30], seven used LED [16, 17, 23, 24, 27, 31, 32], and the remaining two used other light sources [26, 28]. Laser achieved a complete response rate of 63%, and LED achieved a complete response rate of 44%. Laser was found to be more effective in curing oral leukoplakia. When the light source wavelength was > 640 nm, PDT treatment was more effective for the complete remission of the lesion. On the other hand, when the wavelength was ≤ 640 nm, PDT treatment was more effective for the partial remission of the lesion. This may be due to tissue absorbing less light with the increasing wavelength, leading to better light penetration. The optimal wavelength for tissue penetration was 600-850 nm, also known as the “phototherapeutic window” [39–41]. Most studies utilizing lasers for treatment reported side effects or adverse reactions. Patients usually experienced pain, burning, tissue edema, and erythema at the treatment site for a period of time, and some even developed ulcers and loss of sensation. Seven out of the included studies [18–22, 29, 30] mentioned side effects or adverse reactions when using lasers, while five [19–22, 30] cited specific numbers of individuals experiencing these side effects or adverse reactions. Among the 248 patients included in the studies, 163 reported pain or ulcers, 22 experienced photosensitivity, 9 had edema, 9 had erythema, 6 had a secondary infection, and 1 suffered from a superficial burn. However, only two studies [27, 31] using LED reported side effects after treatment, such as pain, edema and ulcers. LED offers several advantages over laser in PDT therapy, including safer use, less thermal damage and lower cost [42]. However, laser can reduce side effects by modifying the dose of light source, the exposure duration, and light transmission. For example, low dose or rhythmic use of light sources may be effective, but these methods are still in the early stages of research and require further investigation [38].

Based on pathological diagnosis, oral leukoplakia lesions can be classified into no dysplastic and dysplastic lesions, with dysplastic as mild, moderate, or severe dysplasia [43]. Out of the 17 studies included in this paper, ten studies statistically analyzed the efficacy of PDT treatment on different pathological types [16, 17, 19–22, 24, 28, 30, 32], among which six studies were evaluated according to unified pathological types [19–22, 28, 30]. After a meta-analysis, it was concluded that PDT treatment could achieve complete remission in leukoplakia diagnosed as no dysplasia, mild dysplasia, and moderate dysplasia, with complete remission rates of 51%, 12%, and 18%, respectively. For leukoplakia diagnosed as no dysplastic and mild dysplasia, partial remission rates were 8% and 15%, respectively. However, PDT treatment was found not to affect severe dysplasia. Severe dysplasia refers to the disorder of cell structure where dysplasia affects more than two-thirds of the epithelial tissue. The architectural disturbances of mild and moderate dysplasia are usually confined to within the middle third of the epithelium and are not accompanied by marked atypia. The lesion tissues of severe dysplasia are deeper than the mild and moderate dysplasia [44]. PDT treatment has limitations. After the local application of photosensitizer, the penetration depth of the light source and photosensitizer may limit the therapeutic effect. For example, the maximum penetration depth of ALA photosensitizer in oral mucosa is 2 mm. Although this method can accurately locate the target tissue, it is difficult to treat deep lesions due to the limitations of penetration depth [22, 45, 46]. Furthermore, this study found that PDT performed better in treating homogeneous leukoplakia than non-homogeneous leukoplakia. It is known that non-homogeneous leukoplakia has a higher risk of malignant transformation than homogeneous leukoplakia, primarily because of epithelial dysplasia. The more severe the degree of epithelial dysplasia, the greater the risk of malignant transformation [47]. Non-homogeneous leukoplakia is more prone to moderate and severe dysplasia than homogeneous leukoplakia, resulting in lower treatment efficacy. These results further confirm the findings of pathological classification.

Follow-up evaluations were conducted a few weeks after the completion of treatment to assess whether the leukoplakia had recurred. Recurrence evaluation criteria were divided into two aspects. On the one hand, clinical observation was performed to determine whether the size and scope of the lesion had expanded or whether new lesions had appeared. This was done using photography and measurements. On the other hand, a histopathological diagnosis was conducted to determine whether there was any further deterioration [48, 49]. The meta-analysis revealed that 13% of leukoplakia patients had recurrence after PDT treatment during the follow-up period. It has been suggested that tobacco smoking, alcohol consumption, and chewing areca nut may lesions be associated with oral leukoplakia’s development and progression [50, 51]. Among the included studies, seven reported on these risk factors [17, 19–21, 24, 25, 32], and five were included in the recurrence analysis [19–21, 24, 32]. Recurrence can be caused by various factors, and modifying these risk factors may reduce the risk of recurrence after PDT treatment.

There are several limitations to this study. Firstly, the number of studies included is relatively small. As a result, there is a significant variation in the quality of the studies, sample sizes, and follow-up durations, leading to increased heterogeneity in the results. Secondly, no statistical analysis of survival outcomes was conducted, which limits the ability to evaluate the long-term effectiveness of PDT treatment for oral leukoplakia. Lastly, the absence of a control group in the single-arm studies analyzed in this paper makes the results less convincing than those from controlled clinical trials.

## Conclusion

In conclusion, this single-arm study shows that PDT may be an effective treatment option for oral leukoplakia, particularly for cases with no, mild, and moderate dysplasia. However, various factors may impact the therapeutic outcome, including the light source, wavelength, and application duration. Our results show that laser as the light source, a wavelength set > 640 nm, and a medium of 5-ALA with an application duration greater than two hours may lead to better efficacy. Unfortunately, clinical controlled trials need to be improved in this study. Further studies are required to evaluate specific parameters, such as wavelength and application time, to determine the optimal treatment plan to improve efficacy while avoiding adverse reactions.

### Electronic supplementary material

Below is the link to the electronic supplementary material.

Supplementary Material 1

Supplementary Material 2

Supplementary Material 3

Supplementary Material 4

## Data Availability

All data generated or analysed during this study are included in this published article and its supplementary information files.

## Abbreviations

PDT: photodynamic therapy
CR: complete response
PR: partial response
NR: no response
ES: effect size
CI: confidence interval
MINORS: methodological index for non-randomized studies
ALA: aminolevulinic acid
YSGG: waterlase
MB: methylene blue
EH: epithelial hyperplasia
LED: laser-emitting diodes
MAL: methy aminolevulinate
PpIX: precursor of protoporphyrin IX
ROS: reactive oxygen species
