# Supplementary material for: Photodynamic therapy (PDT) for oral leukoplakia: a systematic review and meta-analysis of single-arm studies examining efficacy and subgroup analyses
Source: BMC Oral Health. 2023 Aug 13;23:568. doi: 10.1186/s12903-023-03294-3 (PMC10424357; doi:10.1186/s12903-023-03294-3)
Supplement: Supplementary file 2 — Supplementary Material 2 [file 12903_2023_3294_MOESM2_ESM.docx]

**Additional File 2: Table S2. Detailed search strategy in four databases.**

| **Database** | **Search strategy** |
| --- | --- |
| Pubmed | ((("Photochemotherapy"[MeSH Terms] OR ("Photochemotherapy"[Title/Abstract] OR "Photochemotherapies"[Title/Abstract] OR "photodynamic therapy"[Title/Abstract] OR "therapy photodynamic"[Title/Abstract] OR "photodynamic therapies"[Title/Abstract] OR "therapies photodynamic"[Title/Abstract])) AND (("leukoplakia, oral"[MeSH Terms] OR ("leukoplakia oral"[Title/Abstract] OR "leukoplakias oral"[Title/Abstract] OR "oral leukoplakia"[Title/Abstract] OR "oral leukoplakias"[Title/Abstract] OR "leukokeratosis oral"[Title/Abstract] OR " Leukokeratoses, oral "[Title/Abstract] OR "oral leukokeratoses"[Title/Abstract] OR "oral leukokeratosis"[Title/Abstract] OR "keratosis oral"[Title/Abstract] OR "keratoses oral"[Title/Abstract] OR "oral keratoses"[Title/Abstract] OR "oral keratosis"[Title/Abstract]))) |
| Web of Science | 1 TS=(Photochemotherapy) OR TS=(Photochemotherapies) OR TS=(Photodynamic Therapy) OR TS=(Therapy, Photodynamic) OR TS=(Photodynamic Therapies) OR TS=(Therapies, Photodynamic)  2 TS=(Leukoplakia, Oral) OR TS=(Leukoplakia, Oral) OR TS=(Leukoplakias, Oral) OR TS=(Oral Leukoplakia) OR TS=(Oral Leukoplakias) OR TS=(Leukokeratosis, Oral) OR TS=(Leukokeratoses, Oral) OR TS=(Oral Leukokeratoses) OR TS=(Oral Leukokeratosis) OR TS=(Keratosis, Oral) OR TS=(Keratoses, Oral) OR TS=(Oral Keratoses) OR TS=(Oral Keratosis)  3 #1 AND #2 |
| Embase | 1 'photochemotherapy'/exp OR 'photochemotherapy':ab,ti OR 'photodynamic therapy':ab,ti OR 'therapy, photodynamic':ab,ti OR 'photodynamic therapies':ab,ti OR 'therapies, photodynamic':ab,ti  2 'oral leukoplakia'/exp OR 'leukoplakias, oral':ab,ti OR 'oral leukoplakia':ab,ti OR 'oral leukoplakias':ab,ti OR 'leukokeratosis, oral':ab,ti OR 'leukokeratoses, oral':ab,ti OR 'oral leukokeratoses':ab,ti OR 'oral leukokeratosis':ab,ti OR 'keratosis, oral':ab,ti OR 'keratoses, oral':ab,ti OR 'oral keratoses':ab,ti OR 'oral keratosis':ab,ti  3 #1 AND #2 |
| The Cochrane Library | 1 MeSH descriptor: [Photochemotherapy] explode all trees OR (Photochemotherapy):ti,ab,kw OR (Photochemotherapies):ti,ab,kw OR (Photodynamic Therapy):ti,ab,kw OR (Therapy, Photodynamic):ti,ab,kw OR (Photodynamic Therapies):ti,ab,kw OR (Therapies, Photodynamic):ti,ab,kw  2 MeSH descriptor: [Leukoplakia, Oral] explode all trees OR (Leukoplakia, Oral):ti,ab,kw OR (Leukoplakias, Oral):ti,ab,kw OR (Oral Leukoplakia):ti,ab,kw OR (Oral Leukoplakias):ti,ab,kw OR (Leukokeratosis, Oral):ti,ab,kw OR (Leukokeratoses, Oral):ti,ab,kw OR (Oral Leukokeratoses):ti,ab,kw OR (Oral Leukokeratosis):ti,ab,kw OR (Keratosis, Oral):ti,ab,kw OR (Keratoses, Oral):ti,ab,kw OR (Oral Keratoses):ti,ab,kw OR (Oral Keratosis):ti,ab,kw  3 #1 AND #2 |

MeSH: Medical Subject Headings; ti: title; ab: abstract; kw: key word.
