# Supplementary material for: Photodynamic therapy (PDT) for oral leukoplakia: a systematic review and meta-analysis of single-arm studies examining efficacy and subgroup analyses
Source: BMC Oral Health. 2023 Aug 13;23:568. doi: 10.1186/s12903-023-03294-3 (PMC10424357; doi:10.1186/s12903-023-03294-3)
Supplement: Supplementary file 4 — Supplementary Material 4 [file 12903_2023_3294_MOESM4_ESM.docx]

**Forest plots of subgroup**


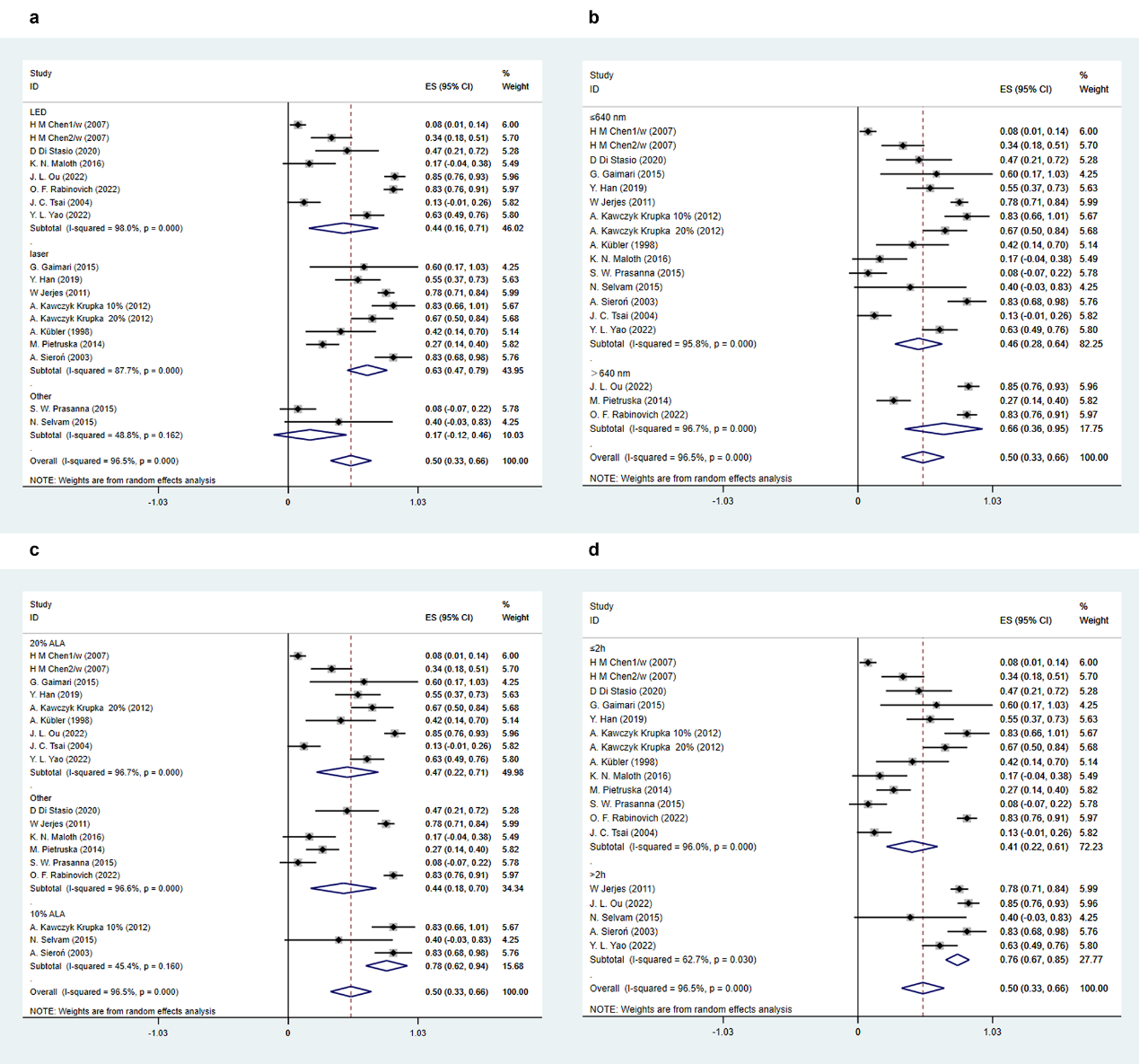


**Figure S1**. Forest plots of CR subgroup according to the influencing factors. (a) Light source. (b) Wavelength. (c) Medium. (d) Duration of application. ES means effect size; CI means confidence interval.


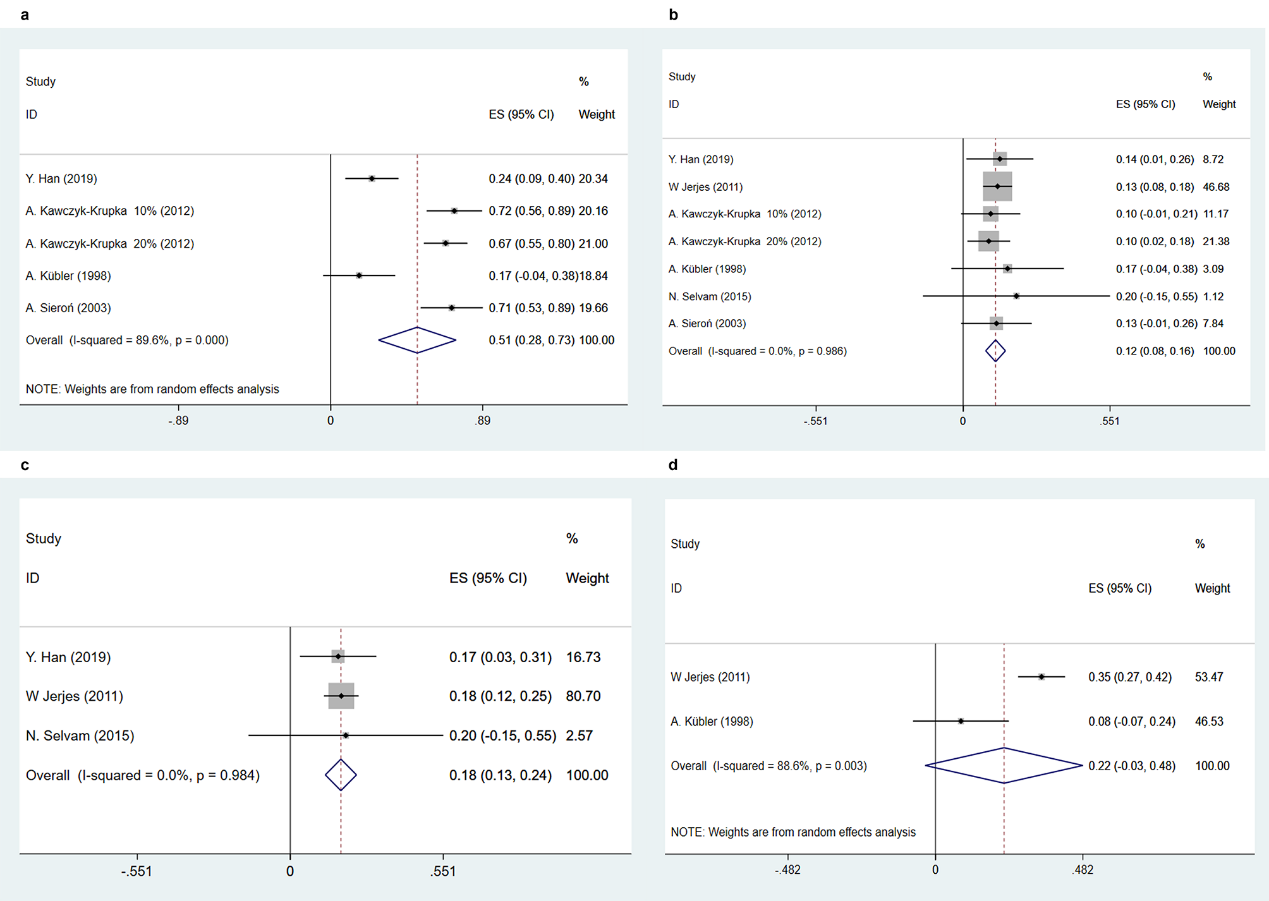


**Figure S2**. Forest plots of CR subgroup according to the classification of pathological diagnosis. (a) No dysplasia. (b) Mild dysplasia. (c) Moderate dysplasia. (d) Severe dysplasia. ES means effect size; CI means confidence interval.


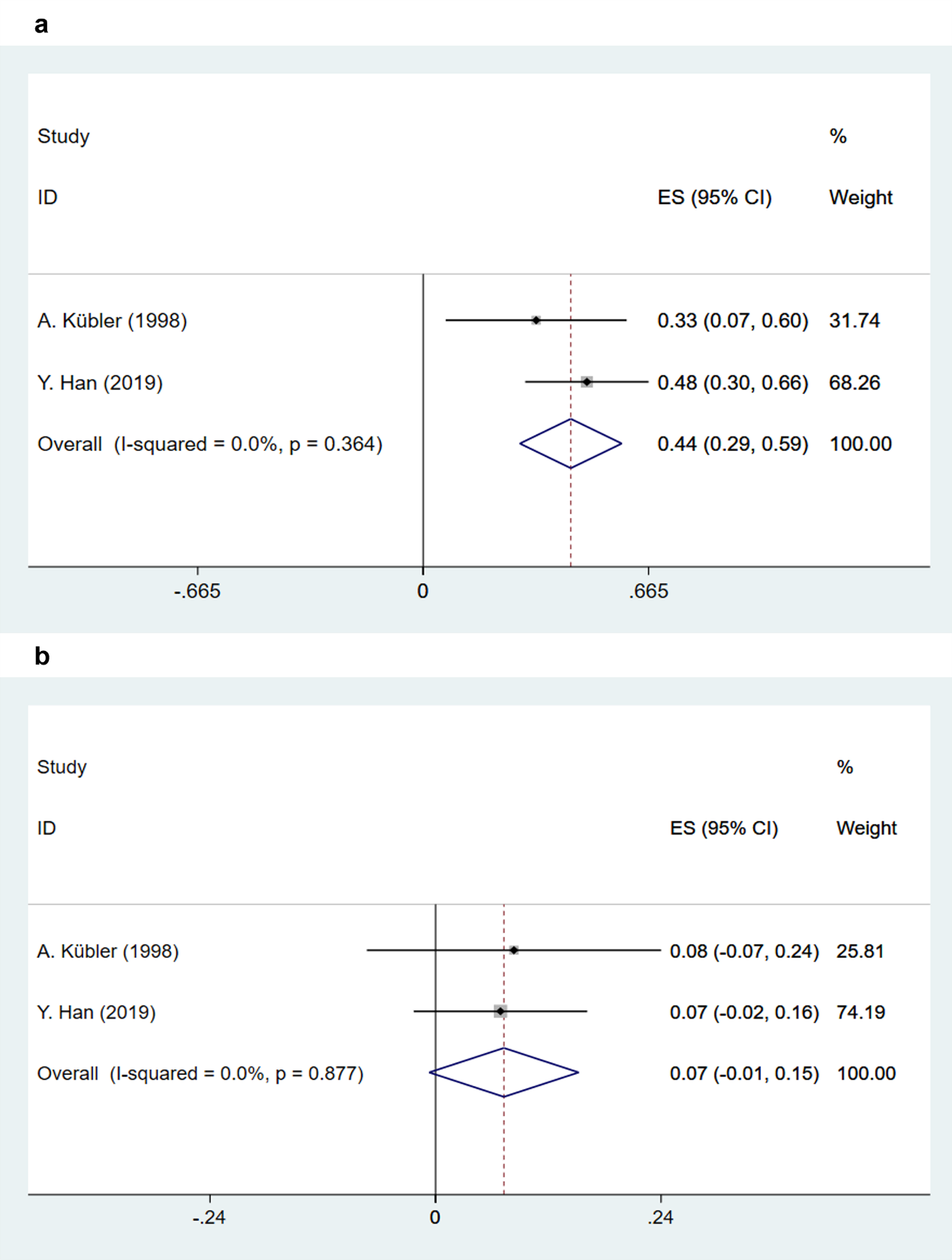


**Figure S3**. Forest plots of CR subgroup according to the clinical classification. (a) Homogeneous. (b) Non-homogeneous.


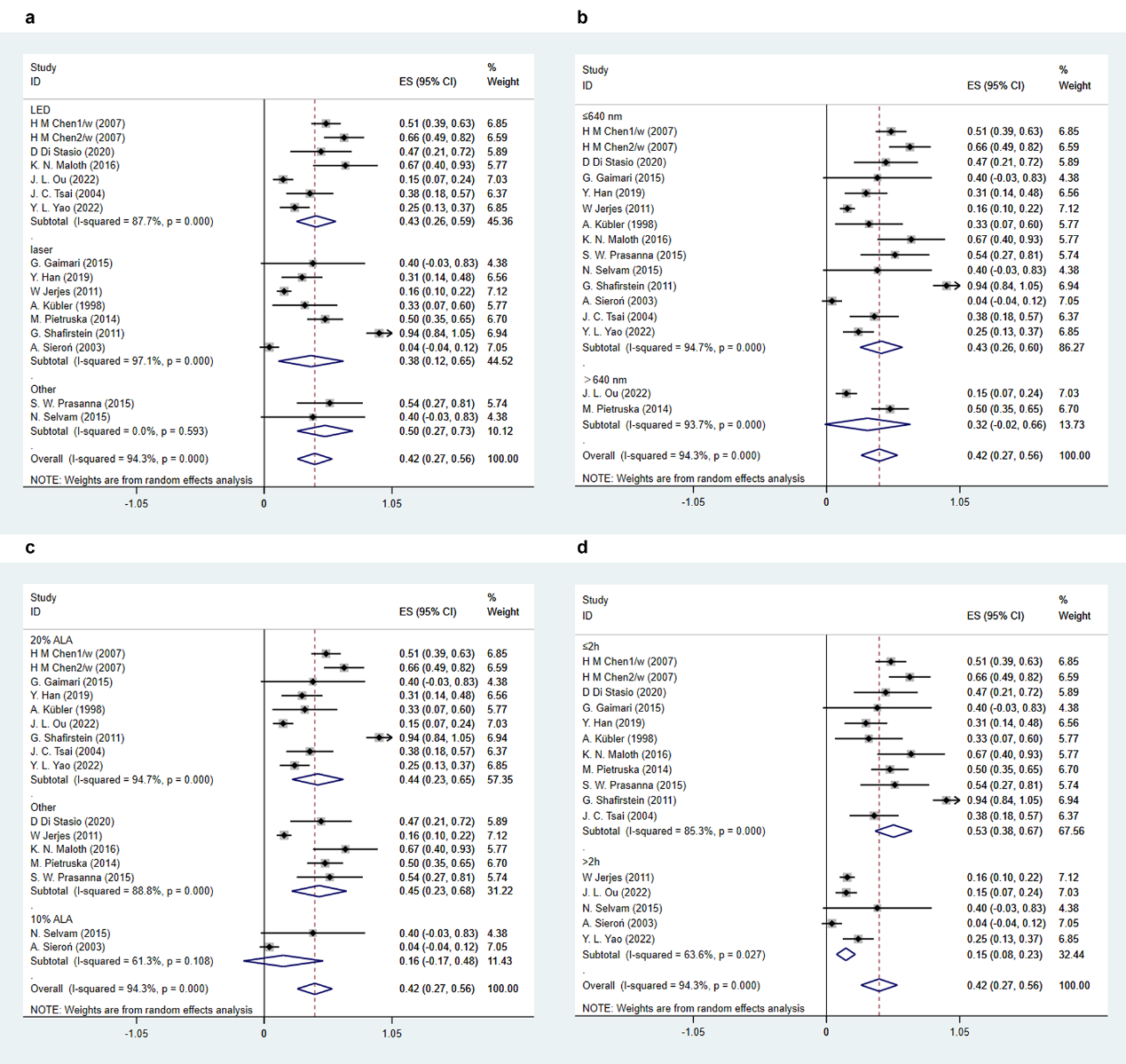


**Figure S4**. Forest plots of PR subgroup according to the influencing factors. (a) Light source. (b) Wavelength. (c) Medium. (d) Duration of application. ES means effect size; CI means confidence interval.


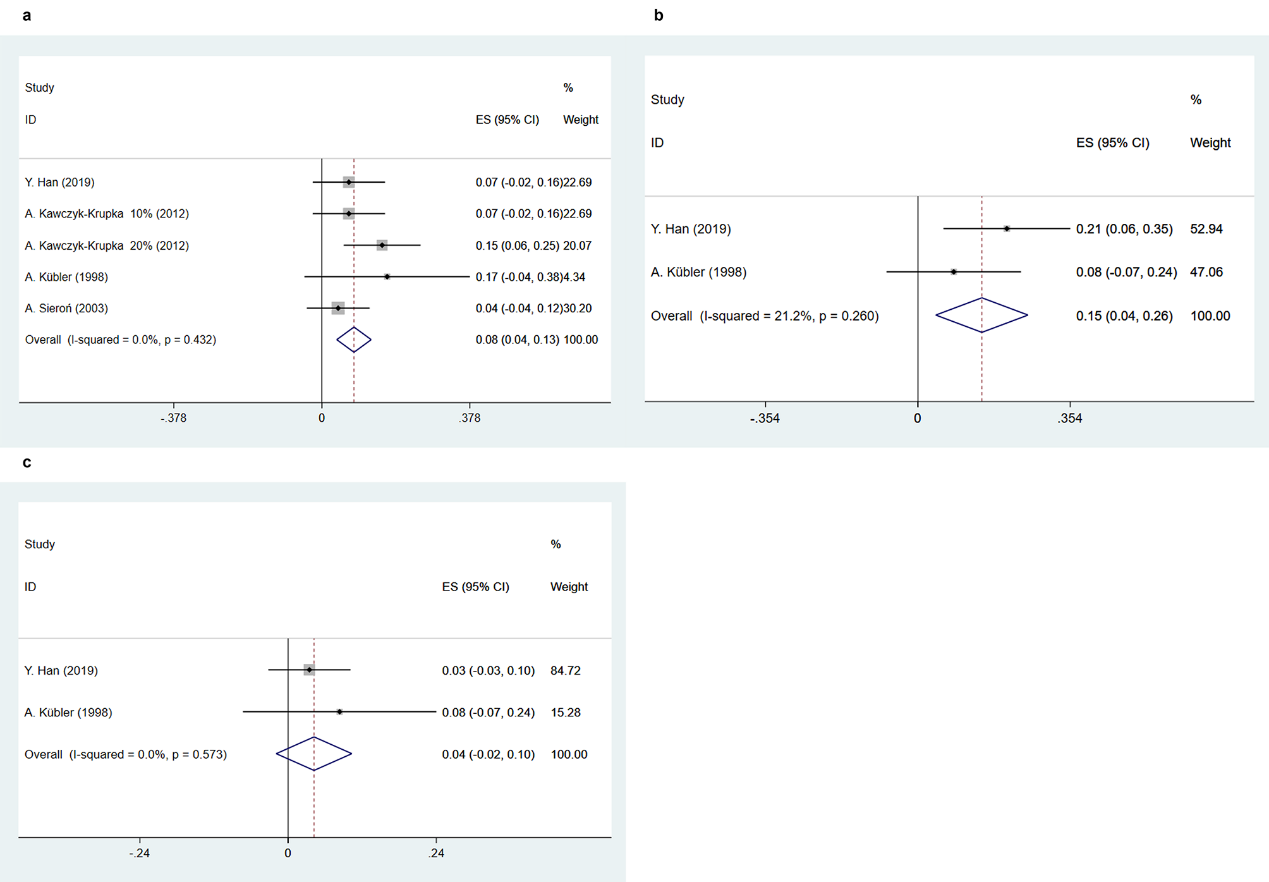


**Figure S5**. Forest plots of PR subgroup according to the classification of pathological diagnosis. (a) No dysplasia. (b) Mild dysplasia. (c) Moderate dysplasia. ES means effect size; CI means confidence interval.


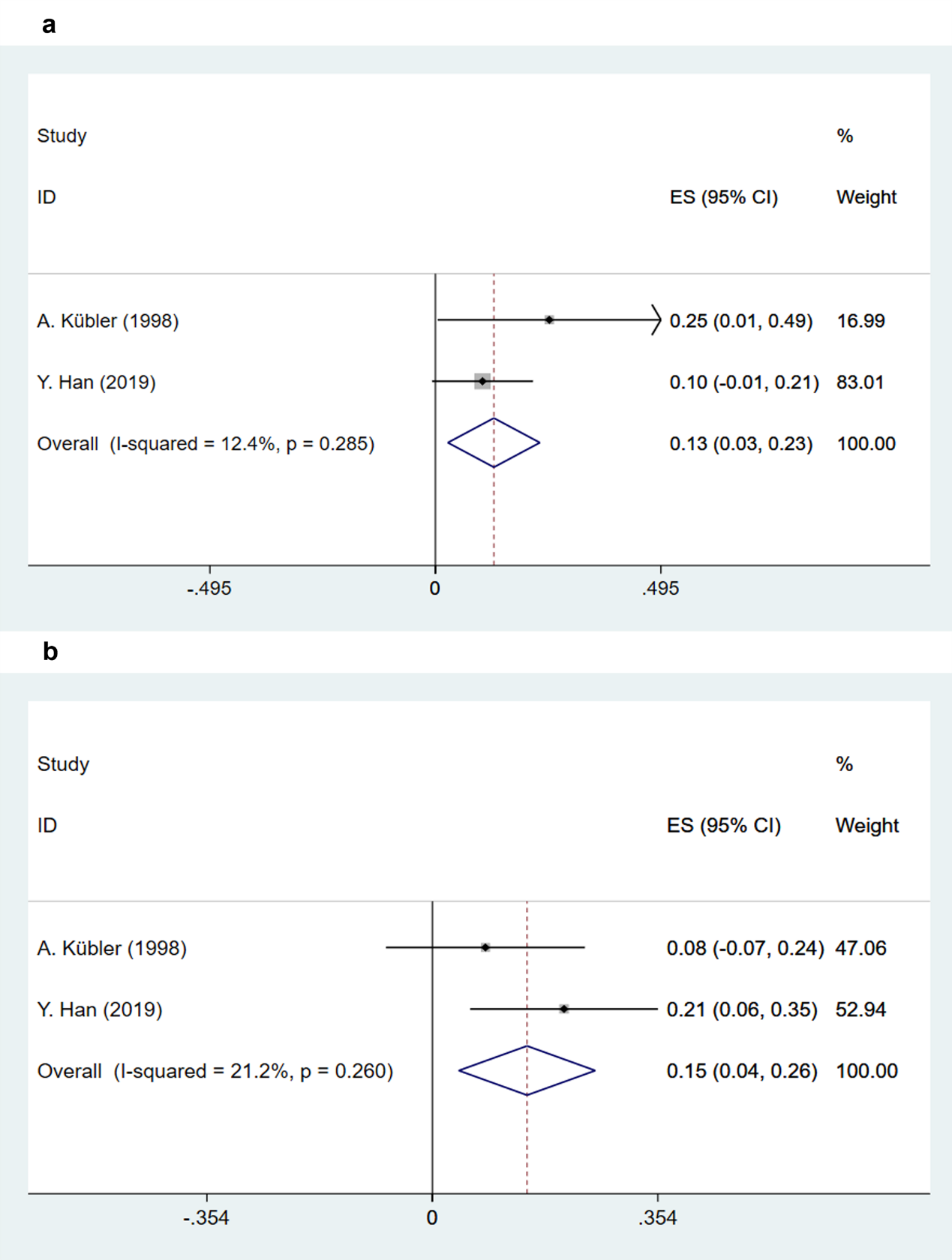


**Figure S6**. Forest plots of PR subgroup according to the clinical classification. (a) Homogeneous. (b) Non-homogeneous.


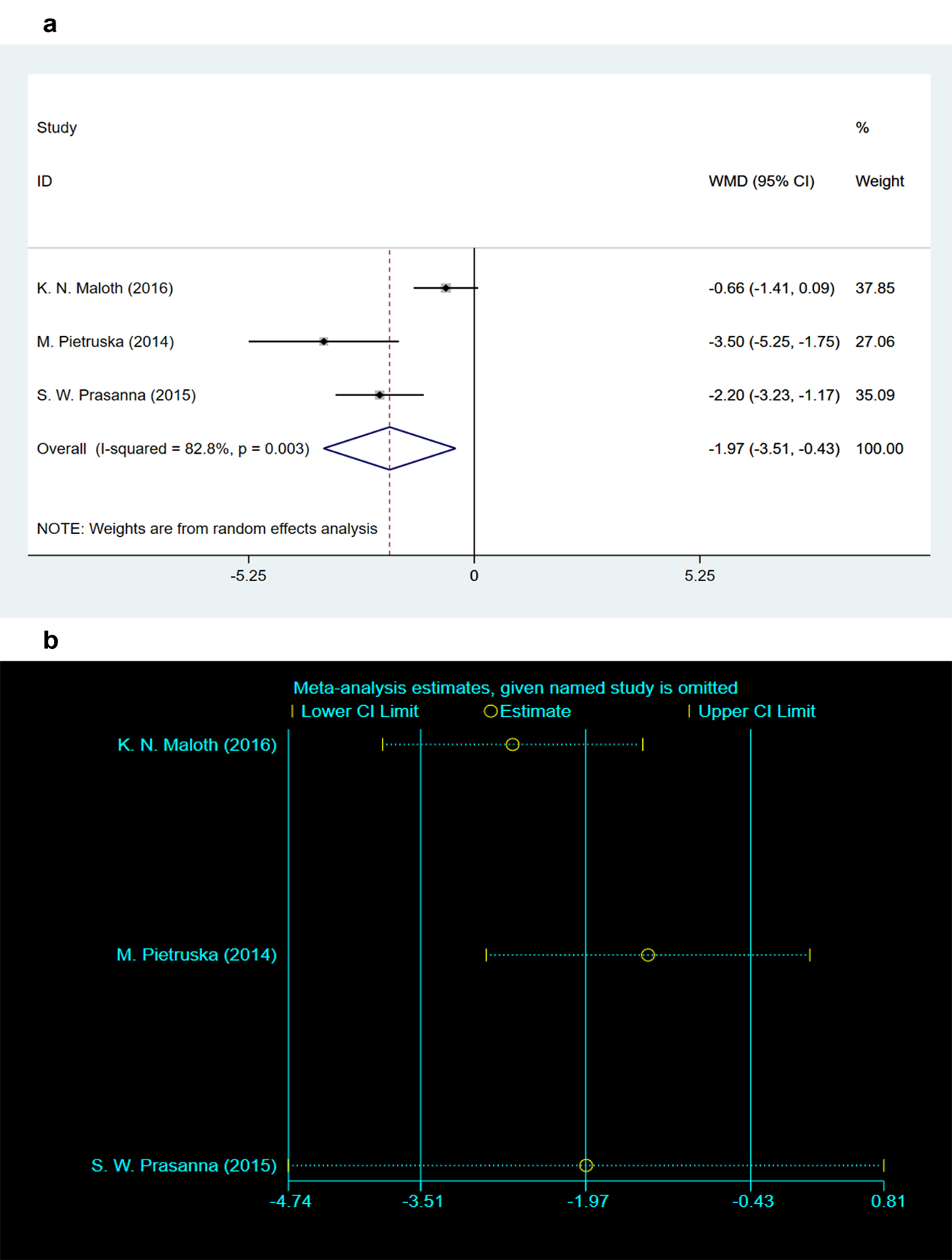


**Figure S7**. Outcomes of the size of leukoplakia after PDT treatment. (a) Forest plot. WMD means weighted mean difference; CI means confidence interval. (b) Heterogeneity analysis diagram. In these studies, no clearly hererogeneous origin could be found.
